# Becoming a Young Breadwinner? The Education, Employment and Training Trajectories of Young Fathers

**DOI:** 10.1017/S1474746415000512

**Published:** 2016-01

**Authors:** Bren Neale, Laura Davies

**Affiliations:** * School of Sociology and Social Policy, University of Leeds E-mail: b.neale@leeds.ac.uk; ** School of Sociology and Social Policy, University of Leeds E-mail: L.Davies@leeds.ac.uk

**Keywords:** Young fathers, parenthood, education, employment, training

## Abstract

The entry into fatherhood is a major life course transition involving the acquisition of new adult roles and responsibilities. This transition is rarely planned for young fathers, and may involve a range of challenges, not least their capacity to provide materially and financially for their child. Drawing on a Qualitative Longitudinal study of young fathers in the UK, this article charts their very different pathways through education, training and employment, showing how these are shaped by a constellation of life circumstances. The implications for policy are considered in the light of a shifting landscape of welfare reform and ‘austerity’ measures.

## Introduction

The entry into fatherhood is a major life course transition involving the fashioning of a new identity and the take up of new responsibilities. For young fathers (aged twenty-five and under, the majority of whom live in socially disadvantaged circumstances (Department for Children, Schools and Families, 2010)), this transition may be unplanned and involve a variety of challenges. Drawing on an ESRC funded study of young fatherhood in the UK, this article explores young fathers’ aspirations to provide financially and materially for their children (the ‘breadwinner’ role). We chart their very different education, employment and training (‘EET’ or conversely ‘NEET’) pathways over time, illustrating how these are linked to their pre-existing socio-economic circumstances. The policy implications of the findings are considered in the light of a normative framework that assumes that early parenthood causes social deprivation, and a welfare framework that regards the needs of young people as a low priority (France, 2008).

### ‘Breadwinner’ ideologies

Providing financial and material support for children has traditionally been seen as the defining feature of fatherhood, while primary caring responsibilities reside with mothers (Roy, 2004). Earning and caring have always been shared by parents to varying degrees, creating a more fluid picture than these traditional models imply, and giving rise to newer ideologies based on a ‘cash and care’ model of parenting (Speak, 2006; Miller, 2011). Nevertheless, the ideological, default framework remains a gendered one. Fathering continues to be mediated through mothers who are gatekeepers to their children. Paid work and provisioning for a household and family continue to be bound up with ideas of adult masculine identity, status and reputation, particularly within working class households (Roberts, 2013). The cultural stereotype of the male breadwinner tends to be reinforced in the UK through a variety of policy and legislative frameworks that take an ‘economic view’ of fathering. Not providing for their family is a key characteristic of the absent ‘bad father’ that can be seen in some UK policies and wider public discourses (Miller, 2011). Whatever factors may lead to refinements in parental roles over time, fathers are likely to start from an imperative to earn, (with some discretion over when, where, and how they care), while mothers start from an imperative to care (with some discretion over when, where, and how they earn) (Neale and Smart, 2002).

Evidence from young fathers suggests a similar balancing of these gendered constructs. While they wish to ‘be there’ for their children and not simply as ‘weekend dads’, they regard breadwinning as a ‘taken for granted’ part of their emerging adult status and identity (Glikman, 2004; Duncan *et al.*, 2010; Negura and Deslauriers, 2010). Whatever their values and aspirations, however, they are likely to face significant social and structural barriers to both caring and earning (Speak, 2006). Their relationships with the mothers of their children and the maternal grandparents are crucial, and yet these are likely to be newly forged and fragile. Moreover, they may have little capacity to provide financial or material (for example, housing) support for their child (Speak, 2006). This is particularly so where they lack formal educational skills and qualifications to propel them into training or employment. Their youthful and dependent status compounds these problems (Neale, forthcoming). Young fathers of post-school age may have fragile EET trajectories that are still very much in the making, while school-age fathers, who enter parenthood before the age of seventeen, are simply too young to either earn a living or access welfare benefits in their own right.

### Employment opportunities and constraints

Becoming a young breadwinner is difficult in the current climate of insecure youth labour markets and reduced welfare entitlements (Neale, forthcoming). In contemporary Britain, only a third of young people with no formal qualifications are in paid employment (Lawton, 2013: 6). A third live in poverty, including those churning in and out of work. Of these, 1.9 million will have an estimated income considerably below the poverty threshold (below 50 per cent of median income). This poverty rate has grown by 6 per cent over the past decade for this age group, and is higher than for any other age group in the UK (Born and Aldridge, 2015).

New Labour's solution to tackling these disadvantages was to encourage and, increasingly, to compel young people into paid work or training. This was the thrust of the ten-year Teenage Pregnancy Strategy, with its unrealistic target to achieve a 60 per cent take up of education, training and employment among young parents (Neale, 2015). The UK has also seen a shift towards increasing welfare conditionality and a drift away from the insurance principles of the post-war welfare settlement. The dominant perception is that unemployment and disadvantage is caused by the characteristics of the unemployed, a deficit of skills and motivation among young people themselves. In the process, the ‘stronger’ explanation for social exclusion, that it is the product of neo-liberal, capitalist economies that rely on a flexible and casual labour force, has been side-lined (Veit Wilson, cited in MacDonald, 2011).

Nevertheless, the rationale that employment is the key solution to the ills of poverty and disadvantage is wearing thin. New dynamic research that moves beyond static understandings of young people as either in or out of work (EET or NEET), is uncovering the fluidity and volatility of employment trajectories over time. This evidence shows that poverty has increased most rapidly among those who churn in and out of temporary work and benefits receipts (MacDonald, 2011; Shildrick *et al.*, 2012). This churning is not merely a feature of early employment experiences, which later settle into something more secure, but is becoming a long-term pattern stretching into later life. Insecurity for young people is rapidly becoming a way of life. As MacDonald (2011) observes, it is *under*-employment that is the deeper and more pervasive problem. For semi-skilled or low-skilled workers, work itself has become ‘poor’: it is sufficiently precarious that it can no longer be relied upon to offer a good enough wage in the here and now, or security for the future.

The effects of these structural changes on disadvantaged young people has been well documented. They face the prospect of precarious EET trajectories; ‘patchwork’ careers in which they churn between training, unemployment and insecure jobs that are low paid and low status, as cleaners, drivers or security guards, on shop or factory floors, or in bars and fast-food outlets. Individuals may be assigned to training schemes in an instrumental fashion that pays scant attention to matching skills and interests with available opportunities, resulting in high drop out rates. They may be subjected to routinised and largely ineffective strategies to find work, for example, cascading their CVs to all employees in an area, regardless of the supply of jobs (MacDonald and Marsh, 2005). Their fortunes are shaped not only by market immobility and precarity, but by a diminishing safety net of security through the benefits system (Simmons *et al.*, 2013). Young people on Job Seeker's Allowance are encouraged to take up of any form of work, regardless of how insecure or poorly paid it is, and, with the threat of sanctions if they do not comply, the process may deskill and impoverish them further (France, 2008).

There is, too, an assumption that young people's needs are less than other age groups, and their wages and benefit entitlements are correspondingly diminished (France, 2008). Two schemes to help young people into work and training, the Future Jobs Fund and Educational Maintenance Allowance (EMA), were axed during the first years of the Coalition government. A replacement scheme, the Youth Contract, was found to be too narrowly focused and ineffective in its design, delivery and impact (Newton, 2014). Towards the end of the last administration, the Coalition chancellor defended plans to remove benefits from young people on the grounds that the imperative was to ensure they were either ‘earning or learning’. The first budget of the new Conservative administration (July 2015) reduced entitlements to housing benefits for young people and announced a living wage available only to those over the age of twenty-five. In this climate, the inequalities gap across the generations looks set to widen further.

It was against this backdrop that we set out to understand the values and experiences of young fathers in navigating their (N)EET trajectories. In the light of a widely reported correlation between young parenthood and social deprivation (Neale, forthcoming), we sought to explore the factors that were making a difference to their life chances, considering to what extent early parenthood is significant in this process, and whether it could be said, in any sense, to ‘cause’ social deprivation. These are contested issues that we return to below.

## Methodology: the following young fathers study

The findings presented here are drawn from two linked ESRC funded studies of young fatherhood, conducted in a Northern city in the UK (2010–15). The research utilised Qualitative Longitudinal (QL) methodology. In the US and Canada this method has been used to generate important insights about young fathers (Glikman, 2004; Negura and Deslauriers, 2010). In the UK, however, there is a paucity of qualitative and longitudinal evidence on young fathers. Our study has generated a unique and extensive dataset that affords new insights into their unfolding lives. We have also filled a significant gap in the current longitudinal evidence base, which is limited in the main to surveys that are tracking older cohorts born in the second half of the last century (for example, Kiernan, 1997). The value of QL research is increasingly recognised in the UK. While longitudinal enquiry turns a ‘snap shot’ of social life into a movie, *qualitative* longitudinal research creates an up-close-and-personal movie, with enhanced depth and explanatory power (Neale, 2015). As Ridge (2015) suggests, this methodology transforms our visions of the social world from monochrome to rich shades of colour and contrast. In particular, it affords unique insights into the factors that shape life course transitions, and the mechanisms that lead to upward or downward trajectories over time (Neale and Flowerdew, 2003; Neale, 2015). It can therefore shed light on debates about the causes and consequences of social hardship.

Current evidence is contested, partly because of the selective use of qualitative evidence in policy (Graham and McDermott, 2005), and because the current longitudinal evidence base upon which to build robust explanations has some limitations. As Neugarten (cited in Neale, 2015) observes, such research does not analyse lives but presents statistical histories of cohorts, with all the attendant dangers of inferring causality from correlations. Those working with large-scale ‘thin’ data acknowledge the inherent complexities of unravelling interactions between individual and structural processes (Such and Walker, 2002; Welshman, 2007: 226–8). These are not either/or factors of course; both are implicated as dynamic processes that are in perpetual interplay as lives unfold. Yet debates may become polarised where analysis rests on over-simplistic, linear models of causality (A causes B). These do not necessarily map onto real world processes, which unfold in complex, non-linear ways. Moreover, linear explanations may feed into simplistic policy responses that may or may not resonate with real lives. Understanding *how* and *why* individual and structural factors intersect to create varied pathways over time requires micro-dynamic evidence that captures *subjective* causality, the nuanced meanings and significance that these processes hold for people themselves. Moreover, by enabling a humanistic approach to unfolding lives, QL research provides a necessary corrective to negative portrayals of marginalised groups in public and popular rhetoric.

Our study has followed the lives of thirty-one young fathers from varied socio-economic backgrounds. Twelve were tracked over five waves of interviews (2011–14), and the remainder over two waves (2013–14) (see details in Neale *et al.*, 2015). Eighteen of the young men were school age fathers, having conceived their first child between the ages of thirteen and sixteen, with most (fifteen) clustered in the age range fifteen to sixteen. The remaining thirteen young men conceived their first child between the ages of seventeen and twenty-four, with a fairly even spread across this age range. Only two of the fathers had planned to enter parenthood at the time that they did, although once they adjusted to the idea their children were far from unwanted. While relationships with the mothers and living arrangements were highly varied, a substantial majority maintained contact with their children over the course of the study. The intensive tracking enabled us to chart the changing fortunes of the young men and capture something of the volatility of their lives over relatively short periods of time. The diverse nature of the sample enabled an exploration of the young men's EET pathways and the causal processes involved in these variations. The prospective longitudinal window provided into their unfolding lives is a relatively modest one, covering between two and four years; the trajectories described here are clearly far from fixed but very much ‘in the making’. Nevertheless, it is possible to discern clear differences in the way these pathways have taken shape and are unfolding, as we report below.

## Findings

### Aspirations and values surrounding breadwinning

In line with existing evidence, the young fathers in our study uniformly held to the values of both ‘earning’ and ‘caring’ as core attributes of fatherhood. Providing for their children was a ‘given’, but the accounts reveal the importance attached to ‘being there’ in a loving, personal relationship with a child. Indeed, these roles were intertwined: being a provider was inextricably bound up with being ‘close’ to a child:

I was only eighteen, so it was . . . a shock, like I said, it forced me to grow up, to take responsibility . . . to go and look for work . . . [A good dad is] one that listens, always there, no matter what. And teach you things . . . bed-time stories and just spending time, that important time. It's not just about buying them clothes, buying them this and that. It's – it runs a lot deeper than that. It's having that bond . . . My son . . . wants to be like me . . . get my hairstyle, support the same football team . . . It's wonderful to see ’cause I never had that . . . I grew up with [my dad] in and out of my life . . . [Their mum] is . . . unemployed . . . Obviously, things like new jumpers for school . . . we’ll go out and get them. Yeah, I think me working full time . . . it can be the difference for her . . . I think it's important to provide ‒ not just financially, but in all the other areas. (Kevin, semi-skilled, aged 24, employed, wave 1)

The young men often spoke starkly about the imperative to fulfil their provider role:

I want any job that's possible, cos if I didn't have money, that’d mean I’m a bad dad ‘cos I’m not making a living or anything. (Jackson, low-skilled, aged 16, unemployed, wave 2)

[I’m on] benefits money ‒ you haven't earned it, so I haven't provided . . . getting up in morning and grafting all day, and then coming home . . . that's providing. I’d much rather be in a job, earning. (Jax, low-skilled, aged 18, unemployed, wave 2)

I wouldn't change Sylvie [daughter] for the world, but maybe I wish I would have waited until I had a job . . . and a little bit more money . . . You need a job just to pay your bills . . . But things happen don't they? (Andrew, low-skilled, aged 16, unemployed, wave one)

However, for low-skilled young men, employment and financial security could be remote goals. School-age fathers lacked funds for basics such as milk, nappies and bus fares to visit their children. They relied heavily on their parents in the short term, saving pocket money and other handouts for essentials, and, in some cases, resorting to criminal activity to fulfil their provider role:

It was hard at first, cos that's when you have to buy everything – a cot and clothes and all that lot. And when I were at school I were selling cigarettes . . . and more [laughs wryly] and that's how I made me money to pay for everything . . . I’ve got basically no money. I’m trying to do good in me life and all that. It's a lot of pressure on me. It does your head in . . . I [can’t] take the kids out, nice places . . . You want the best for your kids. You want ‘em in nice clothes . . .I’m struggling to put decent food on the table . . . Yeah, [finance] does play a lot in it. Definitely, ’cause if you ain't got money, I mean you can love your child and that, can't you . . . now't can beat that love . . . But I mean . . . when it comes to feeding and stuff like that, if you can't do that, then your fucked aren't you, basically. (Callum, low-skilled, aged 19, unemployed, waves 2 and 3)

Such experiences can create a disjuncture between young fathers’ values and aspirations around breadwinning, and what they can achieve. Whatever their age and skills at the point of entry into parenthood, very few young fathers in this study felt they had had sufficient time to consolidate their EET aspirations.

### Education, employment and training: trajectories in the making

Across our sample we discerned significant variation in the EET skills, qualifications and experience of the young men. These were the springboards to their increasingly diverging futures. The differing attributes of the young men, ranging from skilled, to semi-skilled to low-skilled, provides the framework for our analysis. Our reporting relates to the positions of the young men at the close of our fieldwork and the journeys undertaken to arrive at these positions. These EET states are inherently provisional and porous; some individuals were striving to move up a level or prevent slippage down to the level below. In what follows, we chart these upward and downward transitions over time and the ensuing trajectories-in-the-making. In the more detailed cases, we illustrate how these pathways intersect with the young men's unfolding lives as parents and in relation to other domains of experience.

### ‘Skilled’ trajectories

Six of the young men in our study, all from middle income families, were at university. Three had entered parenthood in their early twenties (in two cases as a planned transition within marriage, and with primary caring mothers); two were in their late teens, and one was still at school. While the trajectories of the older fathers were well defined and stable, the younger ones were more circuitous. Two of these were combining their studies with part-time work, as well as some direct care of their children. Jock modified his aspirations to go onto a higher degree because he faced a triple burden of earning, learning and caring that became relentless when his relationship with the mother came to an end. Ben, in contrast, was eighteen and in his first year of university when his child was conceived within a transitory relationship. He considered giving up his degree to find a job, since his course involved a one-year placement abroad. But with support from his parents, and the mother as primary carer, he focused on securing his degree, continued to live in student accommodation, and fitted his caring responsibilities around his studies. In common with all the young men, his child provided an additional motivation to pursue his career:

It's just completely altered my trajectory as it were – it's like a responsibility that's always there . . . I’m really pleased I went [abroad] actually, yeah. Rather than dropping out of Uni and in a substandard job. It felt like . . . you know, the most logical course of action to take. And the one that was kind of most in line with the plan I’d had . . . and then hopefully get a decent job . . . I find it quite a difficult situation because on the one hand I don't have the money to provide, but on the other hand I really do want to be able to provide . . . But it's worth a short period of . . . struggling through it for potentially quite a comfortable existence after university . . . And it would be, ultimately it would be better for [my daughter] . . . In my home town, the twenty-year olds are unemployed and going out and getting drunk. I’m not like that, I’m working towards something better . . . I can still be a good dad and also do what I want. (Ben, skilled, aged 19/20, full-time university student, waves 1/2)

As a final example, Dominic's entry into parenthood at the age of sixteen triggered a disruption to the longer-term, skilled trajectory that he had aspired to while growing up. He managed good grades for his GCSEs, which gave him entry to a 6th form college, but to support his child he combined studying with part-time, semi-skilled office work. During college he separated from the mother and contact with his child was curtailed. His earning power became a critical factor in his efforts to reinstate contact. Like Jock, he was then engaged in a triple burden of earning, learning and direct care of his child, which he managed with the support of his parents. However, these events took their toll and he dropped out from his A-level course. At the time of his first interview, Dominic had moved to full-time office work; he was on a stable, semi-skilled trajectory that did not match his educational profile and aspirations. Four years later, Dominic's life was back on track: he was studying for a degree and with plans for a professional career. He was supported in this by his learning mentor, and his parents, who helped him find a regular pattern for combining degree level study, a part-time job, an unpaid internship and direct care of his child. We trace his journey across two waves of interviews:

I’m not best pleased I’m in this job . . . cause it's really mind numbing . . . So it's not for me . . . I’m purely there just to, just to take money out, cause of financial constraints . . . I have to get some sort of income when it comes to a child . . . Regardless of . . . what I feel I can do, it is just a case of keeping my job . . . Before this happened, you know, I’d, I’d had aspirations of what I wanted to do . . . But I feel quite trapped at the moment, I’m quite aimless. What's the point in having any ambition if you just, you know . . . Everything's a struggle. (Dominic, semi-skilled, aged 18, employed, wave 2)

Going down to part-time from full-time's a massive shift in your lifestyle . . . In some ways, it's lucky how it's turned out where I’ve had three years work experience . . . [I knew] I wasn't going to Uni just for the sake of going . . . I’ve got a direction, it gives me motivation . . . I work evenings so I have the full days to commit to Uni . . . As I say it's very tiring ’cause I’m up at eight . . . and then it's a case of I’m working till ten at night . . . It's all building towards, hopefully, a career path in future . . . I’m genuinely a lot more happy. (Dominic, skilled, aged 21, part-time student/part-time employed, wave 4)

### ‘Semi-skilled’ trajectories

Eleven fathers were in this group at the close of our fieldwork. One had entered parenthood in his early twenties, two in their late teens and the remaining eight were school-age. Six fathers were from middle income families and five from low income families. Relationships with the mothers varied, but these young fathers had maintained a regular commitment to their children over time. Iman, who had been in a fleeting relationship, had an academic identity and background. His aim was to secure a longer term strategic pathway through sixth form college and university, which would take him into the skilled category over time:

It's given me that drive and determination . . . Although I did have my aspirations . . . But it's made me . . . you know, it's made it concrete ‒ the only way I can do that is by studying hard ‒ or winning the lottery overnight [laughs] . . . I’ve explained the bigger picture ‒ you know, I said to [the mum], ‘I could just get a job now, you know . . . stop my studying and just, you know, any money I give it to the kids. But then what is that benefiting the kids? That's just money for now. That money goes. It's not putting nothing in place for them kids. (Iman, semi-skilled, aged 16, full-time student, wave 1)

Kevin, in contrast, prioritised paid work over his construction college course in order to support his child and then partner (he was in this relationship for seven years). With great regret, he left college at the age of eighteen and held several temporary jobs before securing a full-time stock control job at a warehouse. At the time of his interviews with us he was studying for a National Vocational Qualification (NVQ) in warehousing, which would enable him to apply for promotion or a higher paid position:

To come out of college, no real grades from school, not even completed a year of college, I was worried . . . who would employ me? . . . I had four cleaning jobs and I just worked and worked and worked and provided. It was hard, yeah [laughs] . . . looking back maybe I could have stayed at college and got a few years under me. (Kevin, semi-skilled, aged 24, full-time employee, wave 1)

Like Kevin, most of the semi-skilled group were following shorter term EET trajectories, attempting to accrue skills and qualifications through a piece-meal mix of study, training, and low paid, insecure employment, often with more than one part-time job at a time. A key aim for these young men was to secure better work, a pay rise or promotion, or extended or better hours, or improved conditions or a more permanent contract. Indeed it was striking that finding a job was simply not enough; there was ongoing pressure to find a *secure* job, to move from under-employment to full employment. This was a difficult and protracted process, necessitating relentless searching and changes in direction.

If finding the right kind of work was a challenge, so too was securing appropriate training and apprenticeships. Senwe had attended a decorating course on day release from school, followed by a full-time college placement for an NVQ. Unable to meet the standards to progress to the second year, he faced a demoralising period of unemployment, and low-skilled, short-term work in a fast food outlet, through which he was supported by his mother. Motivated by his daughter, mother and connexions worker, he eventually secured a two-year apprenticeship in bricklaying through the Construction Industry Training Board. This ‘snakes and ladders’ process, on the borders of semi- and low-skilled status, took three years:

It's just that challenge of growing up, manning up, knowing that you’ve got a kid coming on the way . . . I was really upset and down, ’cause most of my friends have gone to Uni or college and stuff, doing something with their life . . . and I didn't want to let my mum down as well ‒ just stay at home . . . and do nothing . . . I applied for three apprenticeships and went to an interview. Yeah, I got it! . . . Really happy . . . every day I just try my hardest and work hard so after two years I’ll get the job. Not to just finish with nothing and just go back down on the dole. Now it's less stressful ’cause I’m earning instead of relying on my mum . . . So it's made me a better person to provide for my daughter, and just knowing that I’m working for her. And I feel proud of myself for doing that. (Senwe, semi-skilled, aged 16/19, Apprentice, waves 1/5)

The perseverance of these young men in improving their lot in life was striking; their accounts revealed the sheer hard work needed to secure, sustain and consolidate a breadwinner role in a context where they could not take their EET trajectories for granted. Like Senwe, Richard, Simon and Orlando also moved up from low to semi-skilled positions over time. Through protracted and circuitous routes, including debilitating periods on the dole, they found full-time jobs: in garage and warehouse work and in a sorting office. They saw these as staging posts on the way to the kind of work that they would eventually like to do, and, with good family support, they were sustaining their motivation.

Most of the young men in this group were strongly committed to securing semi-skilled occupations. In many cases, they fitted the traditional model of the working-class fatherhood identity that is centred around the idea of breadwinning for their families. At the same time, these EET trajectories bear all the hallmarks of a casualised and ‘patch work’ labour market, necessitating shorter term decision making and horizons and uncertain futures.

### ‘Low-skilled’ trajectories

Fourteen young men, all from low income families, fell into this category at the close of our study. They had accrued negligible qualifications, skills or experience upon which to build an EET trajectory. Their chances to compete for work in the labour market were slim and their journeys through school and post-school training were chequered, with higher drop out rates for both. Their financial circumstances were often precarious and they were dependent on out-of-work benefits, and with only limited family support for income and resources. Their aspirations to work as an integral part of their fatherhood role were no less strong than those of the other young men in the study, but the gap between aspirations and achievements was more pronounced, both in terms of embarking on a particular path and sustaining it over time:

So I’ve worked . . . a couple of times but it's just because it's always been temporary. So around these times . . . they always lay me off. So it's ‒ I’m always looking again and looking again, until I find something again and I just start right over again. But it's just, it's just really hard to find something permanent this time because I didn’t, I didn't finish my studies well and I didn't do no apprenticeship courses. So it's really got a bit hard for me to go to the, to, to the level of job that I, I was really supposed to, I really wanted to go to. (Marcel, low-skilled, aged 19, unemployed, wave 1)

Nine of these young men were school-age fathers, but, in each case, their engagement with school was tenuous. Some had been excluded or had poor levels of attendance that had impacted on their self confidence and achievements. The young men who had conceived a child later in their teens or early twenties were faring no better. Since they had accrued very few skills since leaving school, their low-skill status was becoming an entrenched part of their credentials and identity.

The strategy of investing in training and qualifications as a more secure route into employment was well known to these young men. Jakie, for example, hoped to gain skills and a qualification in plastering:

If I do get a full-time job . . . it's never 100% guaranteed that I’m always going to have it . . . six months down the line . . . [if] the company has fall backs and has to let people go, I just feel I’m going to be in the same boat again. So I’d rather go and get a certificate saying I can do something and I’ve got more chance of getting a job. (Jakie, low-skilled, aged 23, unemployed, wave 1)

However, as we have seen, tailored routes into training were not easy to secure or sustain.

Alongside their tenuous EET pathways, the young men in this group were grappling with problems in other areas of their lives. Ten of the fourteen fathers disclosed family ‘troubles’ as part of their childhoods, and in their own lives since (and these may have been under-reported among the remainder). Problems ranged from parental drug addiction, prison sentences, mental health problems and physical abuse, to frequent changes of abode, periods in social care and tenuous or volatile relationships within and outside their families. Although the nature, extent and severity of these problems varied widely, the reported correlation between young parenthood and social deprivation was clearly evident for many of these low-skilled young men. Their precarious EET trajectories bore all the hallmarks of their volatile lives.

This group did not progress up the skills ladder during the study; there was a greater likelihood of downward trajectories. Andrew, one of the youngest fathers, revealed the multiple factors that can lead to a downward path, including limited support from his family:

I had to go [to school] on us own. And that's how come I ended up . . . acting an idiot . . . ’cause I thought, obviously, I, I aren't having any help so I’ll just do . . . as I please now. And I . . . look where I’ve ended now. But obviously, I mean I still could . . . change it. But it's just, it's like hard isn't it, really. (Andrew, low-skilled, aged 16, unemployed, wave 1)

When we first met Andrew he was attending a college course for Maths and English GCSEs, and later tried courses in plastering and preparation for the armed forces. He did not manage to complete these courses. Over the next four years, Andrew's circumstances deteriorated significantly. His relationship with his child's mother was fragile and became increasingly volatile. It was not until his last interview with us that he disclosed that he was unable to read and had difficulties with writing, problems that had shaped and constrained his life since his school days. At wave 5, he had been unemployed and in receipt of Jobseeker's Allowance (JSA) for some years. Finding work was a remote dream, although he tried on a daily basis. He had some practical support from his mother and her partner (he was lodging with them, following the end of his relationship). Yet his mother was not able to assist him financially. His JSA had recently been sanctioned; he had missed a letter informing him of an appointment that he needed to attend. He was demoralised and depressed by his precarious situation:

If I got a job, it would change my life . . . I wouldn't mind being a builder or something. But, like I say, I can't read any communications. So it is hard. Like, I see a job going, and I put in for it and ten others who have, like, A for communications obviously will end up with it . . . I’ve handed [my CV] in at Poundland . . . the Market, fish shops, pizza shops ‒ as daft as it seems, everywhere . . . If I got an interview, I think I’d be happy . . . I am going backwards on myself . . . I just look and look and look and never get anywhere . . . I mean people look at us, ‘oh look at him, on JSA. We’re paying his benefits’, but ‘hang on there, you are, but no-one won't hand us any jobs’ . . . It's the worst, most frustrating thing probably ever . . . I’ve had a few problems and me money's been . . . stopped . . . for about three week now. It's a big thing . . . when you don't have any money . . . I can't travel to get anywhere and have a look [for work] . . . I’ve asked me mum but she said ‘no’ she hasn't got it . . . If I don't get any money or anything from anywhere, I’m going to be probably homeless. Sometimes I even think about crying . . . It's really, really hard. (Andrew, low-skilled, aged 21, long-term unemployed, wave 5)

Andrew's relationship with his daughter, Sylvie (which he reflects on above), was of huge importance to him, and his prime motivation to keep searching for work. But, arguably, his fatherhood status, and the need to provide in order to earn his credentials as a father, placed extra pressure upon him, reinforcing the sense of futility and failure that pervaded his account.

Several other young men in similar circumstances had gone further along these downward routes. Adam had become homeless when he could no longer pay his board and lodging at home. Living in poverty on the minimum levels of benefits, he drifted from ‘sofa’ surfing with friends and family into emergency hostel accommodation, where there was no provision for bringing his child. Callum (quoted above) had been on an upward trajectory between waves 1 and 2 of our study, moving from a college course to an apprenticeship. By wave 3, he was struggling financially, with cuts to his EMA, free travel pass and free meal vouchers. By wave 4, his trajectory was downward. He had lost his apprenticeship and had not received payment for his casual employment. With barely enough funds to live on, he resorted once more to selling drugs.

As Andrew's account shows, the increased conditionality of benefits, and the threat or application of sanctions could further destabilise the fragile lives of these low income, disadvantaged young men. It was not uncommon for them to face mental and physical health issues, which added a further layer of complexity and challenge to their parenting efforts. Indeed, the growing discrepancy between their ideals of fatherhood and what they could achieve in practice was, in itself, detrimental to their well being and their social identities and reputations.

## Concluding discussion

### Feckless young fathers?

Our evidence shows a strongly held ideology among young fathers of the value of ‘being there’ for their children and of ‘stepping up’ to a breadwinner role and identity. This was the case regardless of their EET trajectories and skills levels, their relationships with the mothers or their co-resident status with their children. While fatherhood was not planned, parenthood was seen as a positive turning point in their lives, a source of meaning and an opportunity for a renewed focus on their future goals. In line with existing evidence (Duncan, 2007), this finding challenges the view of early parenthood as a social ill. Our findings also attest to the sheer hard work and determination of young fathers in pursuing a breadwinning role, whether studying for a degree, juggling several jobs at a time or the relentless search for casual work. Many young men faced a triple burden of earning, learning and caring that required great commitment and fortitude. Such evidence should dispel any last vestige of the idea that young fathers are feckless.

### A descent into social exclusion?

The correlation reported in many studies between young parenthood and social deprivation was evident for a substantial number of the low-skilled young fathers in this study. Their strategies were short-term and expedient, based on the imperative to earn. Significantly, they lived with an accumulation of daily hardships in which time horizons shrink and fortunes become increasingly fragile. The ability to formulate plans and mobilise aspirations for the future is, arguably, integral to social citizenship, yet for those facing cumulative hardships, this capacity may be eclipsed in the overriding pre-occupation with day-to-day survival. This in itself can lead to downward paths, risky practices and the erosion of health and well being.

However, for a substantial number of young men in our study, the correlation between early fatherhood and social deprivation did not apply. This heterogeneity is important for it warns against blanket assumptions about the characteristics of young fathers and their needs. Those with a steady accumulation of skills were engaged in longer term planning, working with an imperative not so much to earn but to build a livelihood, a trade or career. These were upward, future-oriented and, in some cases, strategically managed trajectories. The young men had a sustained sense of their own identities and confidence in pursuing their paths, borne of family and peer support and a more secure socio-economic base as a spring board for their lives.

Early and unplanned entry into parenthood can certainly affect, disrupt, stabilise or enhance life plans; it is a turning point that triggers changes in perceptions or orientation. It can be the catalyst to grow up a little faster, to ‘make good’, to try out new forms of adult identity, to find work or other sources of income in the shorter term, or to redouble efforts to secure longer term pathways into trades or careers. Yet, while there was some movement within and between our categories over time, the broader trajectories of these young fathers had already begun to form during their childhoods, shaped by a constellation of socio-economic and relational factors. The disruptions or enhancements caused by early fatherhood, then, do not amount to permanent shifts in the direction of a life. They are more likely to create a finessing, refinement or reinforcement of an existing trajectory, one forged prior to the arrival of a child, through a mixture of individual and structural circumstances. As other research has found, it is pre-existing social disadvantage that causes poor outcomes, not young parenthood per se (Kiernan, 2002; Duncan, 2007).

### Policy implications

Government rhetoric that early parenthood is an individual ‘failure’ that determines life chances and leads to poor outcomes is not borne out by our evidence. National level studies indicate that young people who become NEET when they leave school are most commonly those with the poorest support networks and educational records; and they are more likely to continue in this state into their twenties. There is, ‘a growing polarisation between the advantaged and the disadvantaged . . . a social fracturing which would have been unrecognisable in the society of 20 years ago’ (Bynner, 2007: 377‒8). Our evidence accords with this wider picture. Indeed, it suggests a pressing need for comprehensive measures to support and reward the efforts of young fathers to ‘be there’ and provide for their children and to sustain their role as a parent. Providing the structural building blocks to support their transitions into parenthood requires a focused investment in employment pathways and the provision of an adequate minimum wage for these young men. This would benefit young fathers and mothers, their children and wider families. For vulnerable, low-skilled young fathers, tailored professional support, provided earlier in the life course and sustained through the critical years of education and into training would seem to be essential. There is a pressing need, also, to tackle the generational inequalities that arise in a benefits system that favours older people and that serves, perversely, to punish vulnerable young men through a harsh sanctions regime, rather than provide a safety net of social security. Finally, a more robust evidence base, one that takes the perspectives of young fathers more centrally into account, is essential for the development of policies that are in tune with their lived experiences, and that will attend, with some compassion, to their futures.
